# Genetic characterization of MHC class II DQB exon 2 variants in gayal (*Bos frontalis*)

**DOI:** 10.1080/13102818.2014.960787

**Published:** 2014-11-19

**Authors:** Yongke Sun, Dongmei Xi, Guozhi Li, Tiantian Hao, Yuhan Chen, Yuai Yang

**Affiliations:** ^a^ Faculty of Animal Science and Technology, Yunnan Agricultural University, Kunming650201, People's Republic of China

**Keywords:** Gayal (*Bos frontalis*), DQB exon 2, major histocompatibility complex (MHC) polymorphism, peptide-binding sites (PBS)

## Abstract

In the present study, exon 2 of major histocompatibility complex (MHC) class II DQB gene from 39 gayals (*Bos frontalis*) was isolated, characterized and compared with previously reported patterns for other bovidae. It was revealed by sequence analyses that there are 36 DQB exon 2 variants among 39 gayals. These variants exhibited a high degree of nucleotide and amino acid substitutions with most amino acid variations occurring at positions forming the peptide-binding sites (PBS). The DQB loci were analysed for patterns of synonymous (*d*_S_) and non-synonymous (*d*_N_) substitution. The gayals were observed to be under strong balancing selection in the DQB exon 2 PBS (*d*_N_ = 0.094, *P* = 0.001). It appears that this variability among gayals could confer the ability to mount immune responses to a wide variety of peptides or pathogens.

## Introduction

The major histocompatibility complex (MHC) is a genomic region that encodes a wide range of cell surface glycoproteins with central roles in T-cell-mediated immune surveillance.[1] The MHC type I and II molecules reveal pathogen-derived peptide fragments for identification by antigen-specific T cells, causing in their clonal expansion and differentiation into effector and memory cells.[2,3] Class II genes are expressed mainly by antigen-presenting cells of the immune system, which can intake the invaded pathogens, process their proteins into small peptides and reveal bound peptides to MHC class II molecules to actuate CD4 T lymphocytes, thus triggering adaptive immune responses against invaded and specific pathogens.[2,4]

MHC class II genes encode the α and β chains of DR and DQ dimer molecules. The DQ genes are single-copy genes in the mouse, rat, pig and rabbit. However, in humans and dogs, many DQ genes have been characterized, but only one of them appears to be expressed.[5] Similar variability in the number of DQ loci has been reported in ruminants. For cattle, most haplotypes carry duplicated DQ genes [2,6] and it appears that both DQ molecules are expressed.[7] This duplication combined with the polymorphism greatly increases the variation at the cell surface by inter- and intra-haplotype pairing of α and β chains at the time of dimerization. With duplicated DQA haplotypes, inter-haplotype pairings of DQA and DQB molecules form functional restriction elements which are used in preference to the available DR restriction elements to present antigen to CD4 T cells.[8]

The polymorphism found in MHC class II genes, generally, is confined to exon 2, which encodes the peptide-binding sites (PBS) in the β1-domain.[9,10] Exon 2 of each gene encodes for the first domain of the molecule, and the association of the first domains of A and B form a groove for peptide binding. Some sites form pockets which accommodate foreign peptide residues, collectively known as PBS, and are usually more polymorphic than the remaining sites (non-PBS) of the α and β chains.[10] The significance of this polymorphism probably relates to the immune response for an individual, which requires the ability to recognize a variety of peptides or pathogens.[2] There is evidence for the presence of five DQA and five DQB genes (loci) in cattle that exhibit variable degrees of polymorphism and have a large array of haplotypes.[6,11,12]

The gayal (*Bos frontalis*) is a rare semi-wild bovid species found in Bhutan, Bangladesh, India, China and Myanmar.[13] It has a chromosome complement of 2*n* = 58 [14] which has a significant difference with those of cattle (*Bos taurus*, 2*n* = 60) and gaur (*Bos gaurus*, 2*n* = 56). Due to the remoteness of their habitats and due to socio-political and ecological factors, the gayal is one of the least studied ruminants.[15] Furthermore, it has been listed in the red catalogue of threatened species of the International Union for Conservation of Nature and Natural Resources.

Until now, exon 2 of MHC class II DQB gene has been cloned and characterized in the subfamily bovinae animals, including European cattle [*B. taurus* – 6,16–18], zebu cattle [*Bos indicus* – 19,20], hybrid cattle (*B. taurus* × *B. indicus*) and water buffaloes [*Bubalus bubalis* – 21]. For the ‘unique’ gayal, information about the genomic diversity of the MHC gene is needed. Therefore, in the present study, we investigated the genomic diversity of exon 2 of MHC class II DQB gene in gayal. In addition, we compared the polymorphism of DQB gene exon 2 among cattle (*B. taurus*, *B. indicus* and *B. taurus* × *B. indicus*), water buffaloes and sheep (*Ovis aries*).

## Materials and methods

### Gayal genome samples collection

The animals were pastured at the National Jiumudang Stud Gayal Farm, Dulong Town, Gongshan County, Yunnan Province, People's Republic of China. Blood samples were collected randomly from 39 gayals (*B. frontalis*; 28 ♀ and 11 ♂), snap frozen with liquid nitrogen and then stored at −80 °C until analyses. In a previous study, it was confirmed that all animals were pure bred, and not the hybrids of local Yellow cattle (*B. taurus*) and gayal, based on their appearance and analyses of the mitochondrial DNA (mtDNA) control region and the sex-determining region Y gene.[22]

### DNA extraction, DQB gene amplification and sequencing

Genomic DNA was acquired from blood using standard proteinase K digestion followed by extraction with the phenol–chloroform method.[23] The sequences of exon 2 of MHC class II DQB gene were amplified using the primers LA40 (5-ACTGGATCCCCCGCAGAGGATTTCG-3) and LA41 (5-ATAGAATTCACCTAGCCGCTGCCAGGT-3), which were also used to amplify the DQB sequence of cattle [*B. taurus* – 6].

Polymerase chain reaction (PCR) was carried out in a reaction volume of 25 μL, containing 2.0 μL DNA (approximately 30 ng/μL), 0.5 μL of 20 mmol/L deoxy-ribonucleoside triphosphate (dNTPs), 2.5 μL PCR buffer, 1.5 μL of 25 mmol MgCl_2_, 0.75 μL of 10 μmol/L forward primer, 0.75 μL of 10 μmol/L reverse primer, 0.2 μL of 10× Taq DNA polymerase (5 U/μL, Beijing TransGen Biotechnology Co., Ltd., China) and 16.8 μL of distilled water. Thermal cycling parameters were as follows: 3 min at 94 °C, 35 cycles of amplification (45 s at 94 °C, 60 s at 60 °C and 60 s at 72 °C) and finally 8 min at 72 °C. The purified PCR products were sequenced on both strands using an ABI373X DNA analyser (Applied Biosystems Inc.) at the Sango Biotechnology Company (Shanghai, China). Individuals heterozygous for DQB were identified by the presence of two detectable peaks at the same position in the chromatogram. Then, their PCR products were cloned into pMD18-T and sequenced to define the variants by at least five clones.

### Bioinformatic analysis

Clustal X [24] was used for alignment of all nucleotide and amino acid sequences. Phylogenetic and molecular evolutionary analyses were conducted by MEGA version 4.0.[25] The relative frequencies of non-synonymous (*d*_N_) and synonymous (*d*_S_) substitution in exon 2 were calculated using the modified Nei–Gojobori method [26] applying Jukes–Cantor correction.[27] The significance of the difference between these rates was tested with a *Z*-test of selection at the 5% level, whereby the *P*-values were the probability of rejecting the null hypothesis of neutrality (*d*_N_ = *d*_S_).[26] Pairwise nucleotide distances among all MHC class II DQB variants were estimated by the Kimura two-parameter method.[28]

For calculation of the relative frequencies of non-synonymous and synonymous substitution in exon 2 of MHC class II DQB gene, estimation of the Wu–Kabat variability index of the deduced amino acid sequences and analysis of phylogenetic and molecular evolutionary trends, the following sequences from the GenBank were cited: sheep (U07026–U07034), zebu cattle (X79347–X79352, AJ249716–AJ249718 and AJ249896–AJ249898), hybrid cattle (DQ093608–DQ093611 and DQ092799), European cattle (AY368437–AY368455, U77786–U77800, AJ421631–AJ421636, Y18201, D37954, S43263 and U62321), buffaloes (AY699876–AY699887), dogs (AF043147–AF043165) and humans (HE804186, HE804214, HE804220, HE804244 and HE804246).

## Results and discussion

### Identification and characterization of Bofr-DQB exon 2 alleles

Using the primers LA40 and LA41, amplification products 297 bp in length were obtained for all 39 gayals; each contained the complete exon 2 (270 bp) of MHC class II DQB gene. Thirty-six different variants were identified, of which three variants had been reported previously; they were BoLA-DQB*0103, BoLA-DQB*1005 and BoLA-DQB*2301. The nucleotide sequences of DQB exon 2 have been deposited in GenBank with accession numbers JX840412–JX840447. These new variants were assigned variant names according to the BoLA Nomenclature Committee of the International Society for Animal Genetics (http://www.ebi.ac.uk/ipd/mhc/bola/nomen_committee.html).

**Figure 1.**
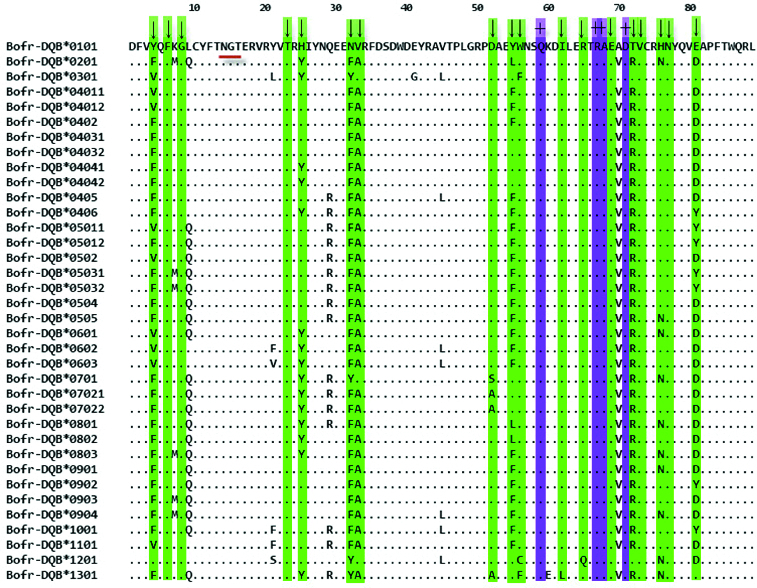
Alignment of the putative amino acid sequences for MHC class II DQB exon 2 from gayal. Dots indicate identity in the amino acid sequence to the sequence of Bofr-DQB*0101, ‘↓’ indicates codons involved in the peptide-binding sites (PBS), ‘+’ indicate the conserved sites about T-cell receptor interaction, N-linked glycosylation site is underlined.

Thirty-six unique amino acid sequences were retrieved from 39 individual gayal (Figure 1). All sequences had N, G and T residues at positions 16, 17 and 18, respectively, which are putative N-linked glycosylation sites and none of the sequences contained deletion, insertion or stop codons, indicating that all sequences found could form functional molecules by modulating various cellular immune responses.[29] We found that 32 of 270 nucleotide sites (11.9%) and 20 of 89 amino acid sites (22.2%) were polymorphic. Among those residues considered important in PBS, 12 (66.6%) of 18 amino acid positions were variable. Most of the variabilities were found at amino acid positions 32, 52, 55, 56 and 81 with three different residues per site, and at position 21 where five residues were observed for gayal.

### Comparison of the genetic diversity of Bofr-DQB exon 2 alleles with other bovina animals

The number of variants from gayal was used to compare their genetic diversity at DQB loci. At present, 80 variants of cattle BoLA-DQB (12 from zebu cattle, 5 from hybrid cattle and 63 from European cattle), 12 Bubu-DQB (from buffaloes) and 9 OLA-DQB (from sheep) are reported in the IPD-MHC database (http://www.ebi.ac.uk/ipd/mhc/). These observations suggest that domestic cattle and gayal have more DQB variants than other species; sheep have the least number of variants. However, the number of variants depends in part on the number of sequenced individuals. It is probable that significantly more domestic cattle have been studied than other species. Therefore, it is still unclear whether European cattle and gayal really have more polymorphic sites at DQB loci. It is noteworthy that in the present study, we found 36 variants in only 39 individuals, indicating the high allelic diversity of DQB loci of gayal.

### Estimation of the Wu–Kabat variability index for Bofr-DQB exon 2 alleles

**Figure 2.**
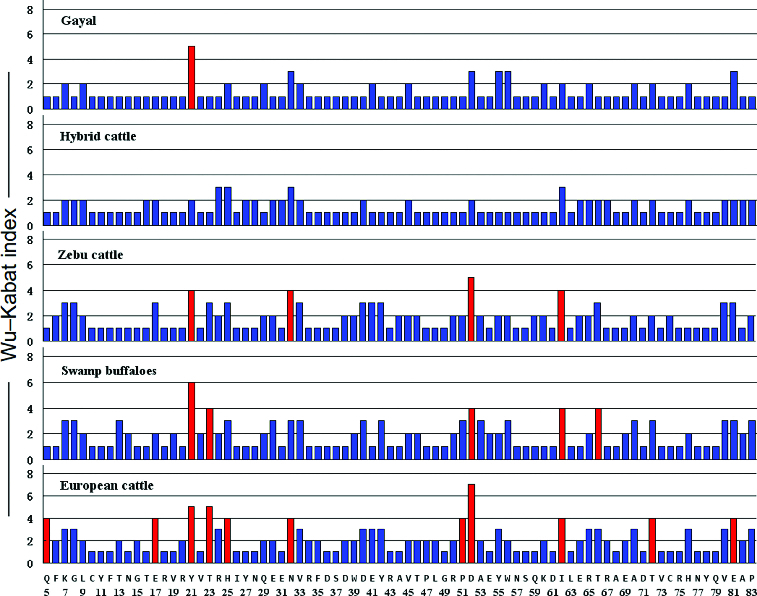
The Wu–Kabat variability index of the BoLA-DQB gene of gayal – *Bos frontalis* (Bofr-DQB), zebu cattle – *Bos indicus*, swamp buffaloes – *Bubalus bubalis*, European cattle – *Bos taurus* and hybrid cattle – *Bos taurus* × *Bos indicus*. The vertical axis indicates the Wu–Kabat index at each amino acid position. The horizontal axis shows the position in the DQB molecules as defined by (5). The consensus amino acid sequence of the DQB molecule is shown below the line.

The Wu–Kabat variability index [30] is a well-established coefficient of the susceptibility of an amino acid position to evolutionary substitutions.[31] It helps to highlight the critical amino acid variation among an array of sequences such as that in the PBS of MHC class I or class II molecules.[32–34] Here, the Wu–Kabat variability index was applied to estimate the coefficient of variation of the BoLA-DQB sequences from the five different bovidae species (Figure 2). An amino acid site with a value of variability of 1 is monomorphic, while a site with a value exceeding 2 is polymorphic. Residue 52 (D) showed the highest variability value in European and zebu cattle. Moreover, European cattle had the highest variability at position 52 where seven different amino acids existed in all variants. Residue 21 (Y) was the second most polymorphic residue, in both buffaloes and gayal, with six and five different amino acids in all variants, respectively. Overall, the DQB gene was most polymorphic in European cattle (11 sites with values exceeding 3) and least polymorphic in hybrid cattle (no sites with values exceeding 3 and only four sites with values exceeding 2).

### High selection pressure for gayal

The frequency of non-synonymous substitutions *d*_N_ was significantly higher than that for synonymous substitutions *d*_S_ in the putative PBS (*P* = 0.001; *t*-test; Table 1). Gayal DQB exon 2 variants appeared to be the product of a locus under high selection pressure for amino acid polymorphisms, because no variant differed by a synonymous substitution (*d*_N_ = 0.094; *d*_S_ = 0). However, minor sequenced samples (39 heads) could have contribution to strong selection pressure.

**Table 1.** Comparison of the rate of non-synonymous (*d*_N_) and synonymous (*d*_S_) substitutions for the peptide-binding sites (PBS) and non-PBS and their ratio among different bovidae species. Standard errors are given in parenthesis. *P*-values are the probability of rejecting the null hypothesis of neutrality (*d*_N_ = *d*_S_).

Taxa	Positions	*d*_N_	*d*_S_	*d*_N_/*d*_S_	*P*-values
Gayal (*Bos frontalis*)	PBS	0.094 (0.024)	0.000 (0.000)	–	0.001
	Non-PBS	0.016 (0.006)	0.014 (0.007)	1.1	0.8
	All	0.031 (0.008)	0.012 (0.005)	2.6	0.06
Zebu cattle (*Bos indicus*)	PBS	0.251 (0.072)	0.054 (0.031)	46.5	0.002
	Non-PBS	0.100 (0.023)	0.136 (0.035)	0.7	0.38
	All	0.128 (0.022)	0.119 (0.028)	1.1	0.8
Hybrid cattle (*Bos taurus* × *Bos indicus*)	PBS	0.181 (0.048)	0.062 (0.072)	2.9	0.2
	Non-PBS	0.098 (0.024)	0.038 (0.021)	2.6	0.07
	All	0.116 (0.021)	0.043 (0.022)	2.7	0.02
Swamp buffaloes (*Bubalus bubalis*)	PBS	0.360 (0.082)	0.101 (0.061)	3.6	0.01
	Non-PBS	0.122 (0.026)	0.179 (0.044)	0.7	0.2
	All	0.172 (0.028)	0.164 (0.038)	1.0	0.9
European cattle (*Bos taurus*)	PBS	0.219 (0.042)	0.064 (0.037)	3.4	0.001
	Non-PBS	0.083 (0.021)	0.067 (0.016)	1.2	0.545
	All	0.098 (0.017)	0.080 (0.020)	1.2	0.47
Sheep (*Ovis aries*)	PBS	0.269 (0.070)	0.062 (0.043)	4.3	0.005
	Non-PBS	0.098 (0.025)	0.082 (0.027)	1.2	0.5
	All	0.126 (0.022)	0.077 (0.023)	1.6	0.06

We interpret the higher ratio of *d*_N_ to *d*_S_ and the high allelic diversity observed in PBS of gayal as evidence for balancing selection on the DQB locus for maintaining high allelic variability. Furthermore, a comparison of *d*_N_/*d*_S_ in the PBS among the different species, as summarized in Table 1, is considered to reveal a similarly high *d*_N_/*d*_S_ ratio, except for hybrid cattle (*P* = 0.20; *t*-test). However, for amino acid sites outside of the putative PBS, all species were found to be under neutral selection. For amino acid sites of the complete exon 2, the high ratio of non-synonymous to synonymous substitutions was observed only in hybrid cattle (*P* = 0.02; *t*-test). The high ratio revealed in bovidae is considered to be the result of a generally very low rate of synonymous substitution in exon 2 and an excess of non-synonymous substitutions. The polymorphic amino acid sites of both DQB are strongly concentrated in the PBS. These highly polymorphic features of DQB in bovidae are considered to be the result that the peptide recognition functions of DQ molecules in bovidae species are more strongly resembled than those of the DR molecules. However, it does not occur in other species except for cattle.[31] Moreover, these results could provide a hint that semi-wild gayal maybe has strong disease resistance when they are occurred from wild to domestic process.

**Figure 3.**
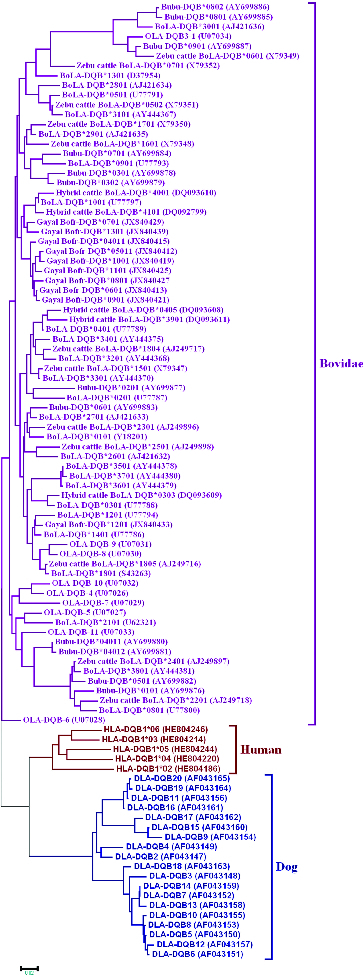
Phylogenetic relationships between DQB sequences for humans (*Homo sapiens*), sheep (*Ovis aries*), European cattle (*Bos taurus*), hybrid cattle (*Bos taurus* × *Bos indicus*), zebu cattle (*Bos indicus*), buffaloes (*Bubalus bubalis*) and dogs (*Canis lupus familiaris*). The phylogenetic tree was constructed using the neighbour-joining method and was based on the nucleotide sequences.

The presence of various variants at a particular MHC locus is also an evidence of the long-term evolutionary persistence of the locus. This is confirmed by the fact that the variants in one species often are related more closely to the variants in closely related species than to the other variants in the same species. This phenomenon is referred to as ‘trans-species polymorphism’.[31,35] When we compared the BoLA-DQB variants with the DQB variants of humans and dogs by constructing a phylogenetic tree using the neighbour-joining method, we found that the DQB variants from zebu, European and hybrid cattle and buffaloes form a single large clade including sheep (Figure 3). This phenomenon is also found in DQA1 locus [36–38] which is consistent with the existence of trans-species polymorphism in bovidae.

## Conclusions

In the present study, exon 2 of MHC class II DQB gene was isolated and characterized in gayal for the first time. Thirty-six new variants were detected. Allelic diversity was analysed and compared with other bovidae species and the PBS of detected variants were confirmed to be under strong balancing selection for gayal.
